# A Recipe for a Good π. How to Properly Estimate Population Genetics Summary Statistics and Why we Should Systematically Report Them

**DOI:** 10.1093/gbe/evag103

**Published:** 2026-06-05

**Authors:** Maxence Brault, Thomas Brazier, Alexander Mackintosh, Anastasia Paupe, Martin Lascoux, Sylvain Glémin

**Affiliations:** CNRS, ECOBIO (Ecosystems, Biodiversity, Evolution), University of Rennes, Rennes, France; CNRS, ECOBIO (Ecosystems, Biodiversity, Evolution), University of Rennes, Rennes, France; Department of Ecology and Genetics, Evolutionary Biology Center, Uppsala University, Uppsala, Sweden; Department of Bioinformatics and Genetics, Swedish Museum of Natural History, Stockholm, Sweden; CNRS, ECOBIO (Ecosystems, Biodiversity, Evolution), University of Rennes, Rennes, France; DECOD (Ecosystem Dynamics and Sustainability), INRAE, Institut Agro, IFREMER, Rennes, France; Department of Ecology and Genetics, Evolutionary Biology Center, Uppsala University, Uppsala, Sweden; CNRS, ECOBIO (Ecosystems, Biodiversity, Evolution), University of Rennes, Rennes, France; Department of Ecology and Genetics, Evolutionary Biology Center, Uppsala University, Uppsala, Sweden

**Keywords:** population genetics, summary statistics, genetic diversity, comparative analyses, FAIR principles, variant calling format

## Abstract

Many long-standing questions in population genomics can now be addressed through comparative analyses and by leveraging the vast amount of genomic data being generated. In the context of questioning the utility of producing such a large amount of genomic data, whether for ecological or economic reasons, we argue that data publication should be standardized to ensure long-term reusability. Based on a literature review and key examples, we emphasize that despite the growing volume of available data, the lack of methodological documentation and the absence of metadata make most published polymorphism datasets incomparable, preventing the calculation of meaningful statistics and the application of FAIR (Findable, Accessible, Interoperable, Reusable) principles. We stress that the Variant Calling Format (VCF) as it is used and published today is insufficient, as it does not report the number of monomorphic sites, which are required to compute basic statistics such as pairwise nucleotide diversity (π) or Watterson's θ. We further propose guidelines and best practices to provide sufficient information to allow the proper calculation of these statistics while accounting for sources of bias and misestimation frequently observed in the literature. Finally, we underscore the need for the systematic reporting of standardized statistics, coupled with transparent documentation of data processing steps, to ensure the reproducibility and comparability of population genomic research.

Significance statement Population genetics relies on fundamental summary statistics, such as the nucleotide diversity (π), to quantify genetic variation within species and address key questions in evolutionary biology, from Lewontin's paradox to the efficacy of selection. However, in the genomic era, π and other related statistics are often missing, inconsistently reported, or severely biased. This stems from the use of variant-only data formats and heterogeneous filtering approaches, which obscure the true number of callable sites and bias estimates of absolute summary statistics. This perspective shows that these methodological issues can introduce orders-of-magnitude discrepancies across studies. To enhance reproducibility and align with FAIR principles, we propose straightforward and actionable guidelines: standardized π estimation, systematic reporting of summary statistics, and the adoption of richer metadata. Implementing these practices should ensure that population genomic data remain comparable and reusable across a wide range of empirical systems.

## Introduction

Since the development of high-throughput genetic sequencing technologies, population genomic data have become available for almost any kind of species. This allows addressing or re-addressing basic questions in evolutionary biology in a comparative framework about the determinants of genetic diversity (Romiguier et al. 2014; Corbett-Detig et al. 2015; Chen et al. 2017; Mackintosh et al. 2019; Buffalo 2021), the efficacy of selection and adaptation at the molecular level (Galtier 2016; Chen et al. 2020, 2022; Leroy et al. 2021), genomic consequences of speciation (Roux et al. 2016; Monnet et al. 2025) and domestication (Arnoux et al. 2021) or variation of past demography among species (Nadachowska-Brzyska et al. 2015; Walton et al. 2021).

To carry out such comparative population genomic analyses, two main approaches are possible: either to directly generate the required data (e.g. Romiguier et al. 2014, Mackintosh et al. 2019, Leroy et al. 2021) or to compile publicly available ones (e.g. Corbett-Detig et al. 2015; Chen et al. 2017; Buffalo 2021). Although generating new data targeted to the question of interest is the gold standard for reliable comparisons among species, reusing already available data has many benefits, such as gathering larger datasets, saving time and money, and limiting the environmental impact of data acquisition and treatment. With the second strategy, statistics of interest can be compiled from initial publications (e.g. Buffalo 2021) or raw data can be re-analyzed (e.g. Chen et al. 2017). Here again, two main options are possible: either to start from Single Nucleotide Polymorphism (SNP) datasets or from scratch by re-processing raw reads. Similar to generating new data, starting from reads offers more standardized and comparable outputs, but it also requires far more time and computing resources. Using genotype/SNP datasets thus appears as a good compromise since, in theory, it offers all the required information to re-analyze the data to address new questions. Despite a more limited scope, basic population genomic statistics (e.g. nucleotide pairwise diversity (π) or Fst) can also be useful for meta-analyses. They provide a first connection between genome sequences and population processes and a global context in which to interpret the results and inform on the biology of the studied species.

In this perspective, we review and discuss the possible issues with the computation of standard statistics in population genomics, how they are reported, and how datasets are made available for re-analysis. We find that statistics and data reporting are highly heterogeneous among studies, both in quality and quantity, which can hinder the applications of FAIR principles (Findability, Accessibility, Interoperability, and Reuse) for reproducible science and bias or even prevent comparative analyses. We propose simple recommendations in reporting information that could be adopted by the community to improve the FAIRness of future population genomic research.

## Standard Statistics Should be Routinely Reported

Basic population genetics summary statistics (e.g. π, θ_W_, Tajima's D, F_ST_, D_XY_) are estimators of population-scale parameters that are easy to report and interpret. Their compilation and comparison among species are often a first key step to address classical (e.g. the Lewontin's paradox) and practical questions (e.g. the quantification of the loss of genetic diversity) in evolutionary and ecological genetics. Beyond their clear utility for comparative analyses, reporting basic summary statistics has at least two other significant benefits. First, it is a simple confidence check of data quality. Basic statistics summarize the raw data with a low level of abstraction, enabling intuitive interpretations and early detection of data issues, such as technical artifacts (e.g. hidden paralogy, centromeric regions, chromosome inversions) or filtering issues (e.g. missing data), before they impact downstream analyses (Dallaire et al. 2023; Jaegle et al. 2023; Tjeng et al. 2025). For example, an excess of genetic diversity in the self-fertilizing plant *Arabidopsis thaliana* showed evidence of pseudo-heterozygotes due to unfiltered hidden paralogy (Jaegle et al. 2023). Second, summary statistics provide useful guidelines for interpreting the results of more elaborate analyses, as the predictions and the power of analyses may vary with the levels of genetic diversity or population structure. Importantly, systematically reporting summary statistics is also a way to develop intuitions for the biological orders of magnitude of genetic diversity, and it could be especially useful for trainee researchers to rapidly acquire a sense of what is a realistic expectation of π, F_ST_, or other statistics for a given species or group of species.

**Fig. 1.**
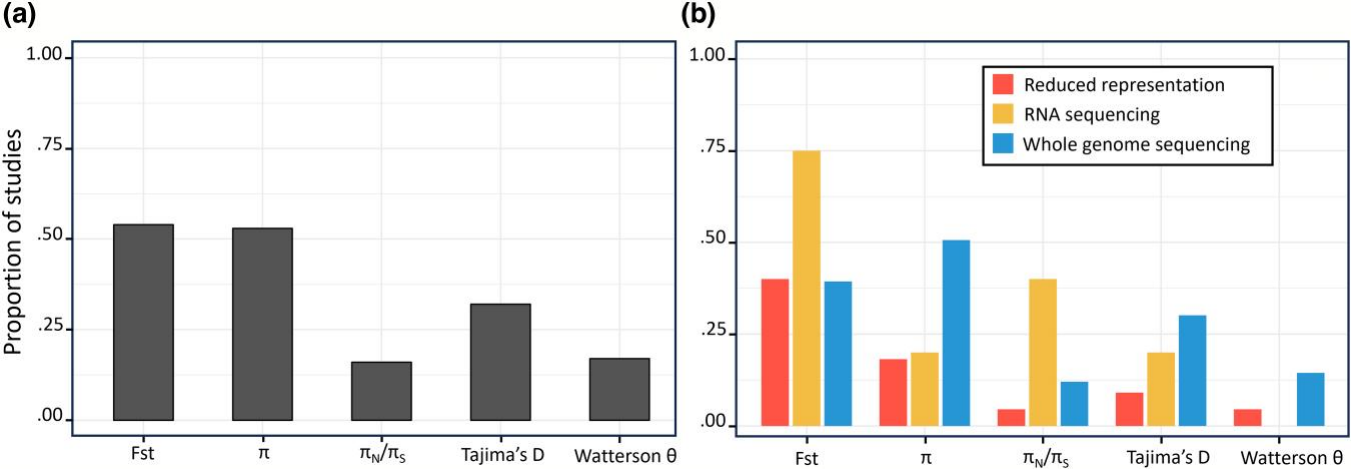
**Proportions of studies published in GBE since 2009 reporting statistics of interest**. a) Proportion of studies reporting summary statistics among 67 studies where Fst is relevant and 90 studies where other statistics are relevant. b) Proportion of studies reporting summary statistics function of data type among 22 papers for Reduced representation (20 for Fst), 5 papers for RNA sequencing (4 for Fst), and 83 papers for WGS (61 for Fst). See Text S1 and Table S1 in Supplementary Materials for the selection of the papers.

Yet, despite their apparent simplicity and universal nature, basic summary statistics are unfortunately often not reported. Interestingly, while such statistics were routinely reported when molecular population genetics was restricted to the analysis of a small number of genes, this practice has faded away with the development of high-throughput sequencing technologies. To illustrate this, we assessed how frequently common population genetics statistics have been reported in the literature using articles published in GBE since its origin, 2009 (Fig. 1, Table S1). We identified 113 relevant population genetics studies (see Table S1 and Text S1). Approximately half of these studies reported the standard estimator π, while the alternative Watterson's θ was reported in 17% of them. The ratio π_N_/π_S_ (or π_0_/π_4_) was often reported for RNA-seq data (40%) but rarely for WGS data (14% in total). Among 90 studies where several populations were analyzed, 36 (54%) reported F_ST_ values. Globally, summary statistics are neither sufficiently reported nor easy to find, and frequently impossible to compare (Fig. 1, Table S1).

Paradoxically, it currently appears more difficult to perform a meta-analysis on diversity statistics than a few decades ago. Earlier meta-analyses, such as Leffler et al. 2012, were limited by a small number of sampled individuals and few genes (only 24 of 280 estimates were chromosome- or genome-wide), but substantially benefited from a more consistent reporting of values. In contrast, in modern genomic studies, basic summary statistics are often substituted by more sophisticated representations such as windows-based estimates, graphical representations (e.g. Manhattan, Circos or Structure plots) or direct output of inferred parameters (e.g. directly reporting estimates of effective sizes and divergence times). This strongly limits their utility for broader scientific re-use and meta-analyses since genome-wide values are almost impossible to infer from them. This is well illustrated in a recent reappraisal of Lewontin's paradox (Buffalo 2021) that leveraged estimates of genetic diversity up to 2015, mostly from Sanger sequencing of a few genes from Leffler et al. (2012) or synonymous diversity in transcriptomic data (Romiguier et al. 2014), leaving aside recent WGS data from the last decade.

## Standard Statistics Should be Properly Computed

**Fig. 2.**
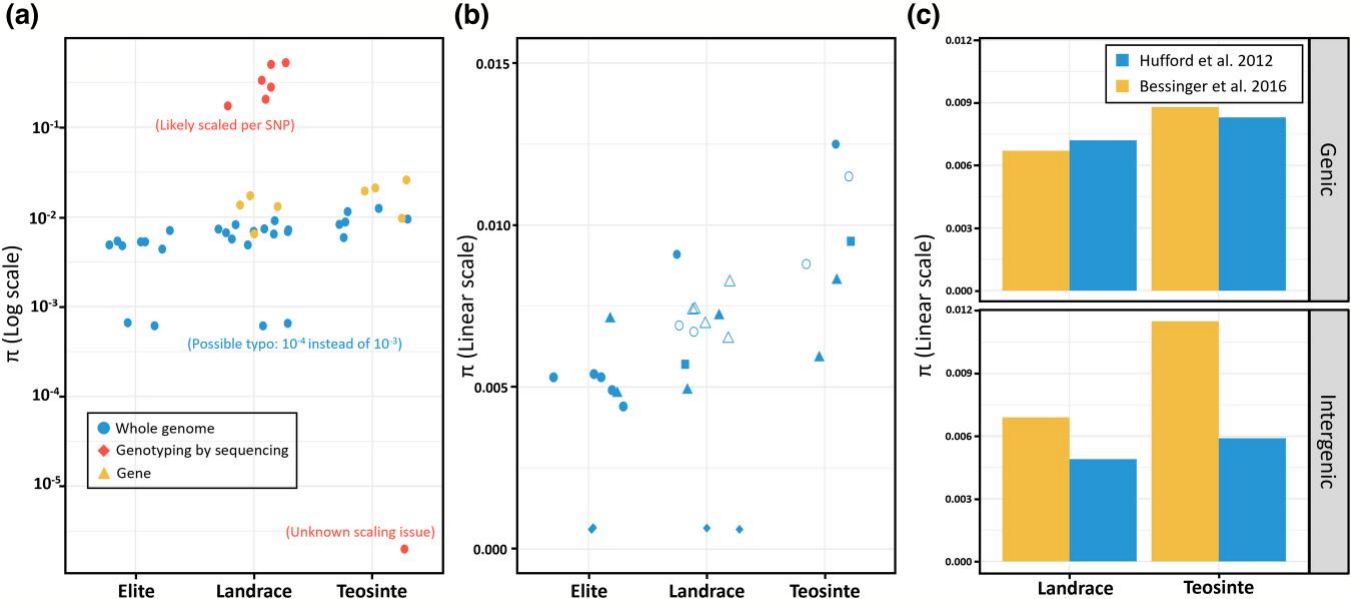
**Distribution of π estimates in 15 population genetics studies in maize, published from 1998 to 2025** (*n* = 43 estimates, Table S2, text S1). a) Distribution of π estimates across elite lines, landrace populations and the wild relative teosinte. Colors represent the sequencing approach. b) Distribution of π estimates among WGS studies. Each study is represented by a specific dot shape. c) Comparison of the genome-wide average of θπ between two similar studies, calculated on genic or intergenic regions.

In the previous section, we highlighted that summary statistics are reported infrequently. Furthermore, when they are reported, heterogeneity in genotype filtering and calculation methodology limits the reliability and reusability of estimates. To illustrate this issue, we retrieved and compared π estimates across 15 studies on maize, which has been studied for decades and for which levels of genetic diversity in wild and cultivated populations are well known. Surprisingly, reported π estimates span an implausibly large range, from 2 × 10^−6^ to 0.523 (Fig. 2, Table S2), which is more than two orders of magnitude larger than what is observed among all Metazoans (Buffalo 2021).

This survey highlights the main issue in computing π, which is caused by the almost ubiquitous use of variant-only VCF files. In contrast with alignment format (e.g. BAM), the original VCF format (Danecek et al. 2011) cannot distinguish true invariant sites from missing data. Information is thus missing to properly estimate standardized absolute statistics, such as π or D_XY_ (Korunes and Samuk 2021; Konopiński 2023; Bailey et al. 2025; Mirchandani et al. 2025). For GBS data, normalization is often done by the number of polymorphic sites, which explains the very high values in Fig. 2 (see also Text S2). To avoid confusion, such statistics should not be reported as π and authors must be explicit about the statistic measured. Another common practice is to standardize statistics by the total length of the reference genome, implicitly assuming no missing data for invariant positions. This is well illustrated on Fig. 2c (see also Box 1) where the estimates of π are very similar for genic regions of the maize genome but surprisingly different for the whole genome estimates. π was likely scaled by the total reference genome size in Hufford et al. 2012 instead of the size of the callable part of the genome as in Beissinger et al. 2016, with larger differences in intergenic regions that exhibit more missing data. There are other subtleties in computing statistics with missing data, yet, properly calculating the denominator (effective number of observed sites) drives the far largest, consistent reduction in bias across methods, as illustrated by Korunes and Samuk (2021, their Fig. 3).

Box 1. Toward an unbiased π

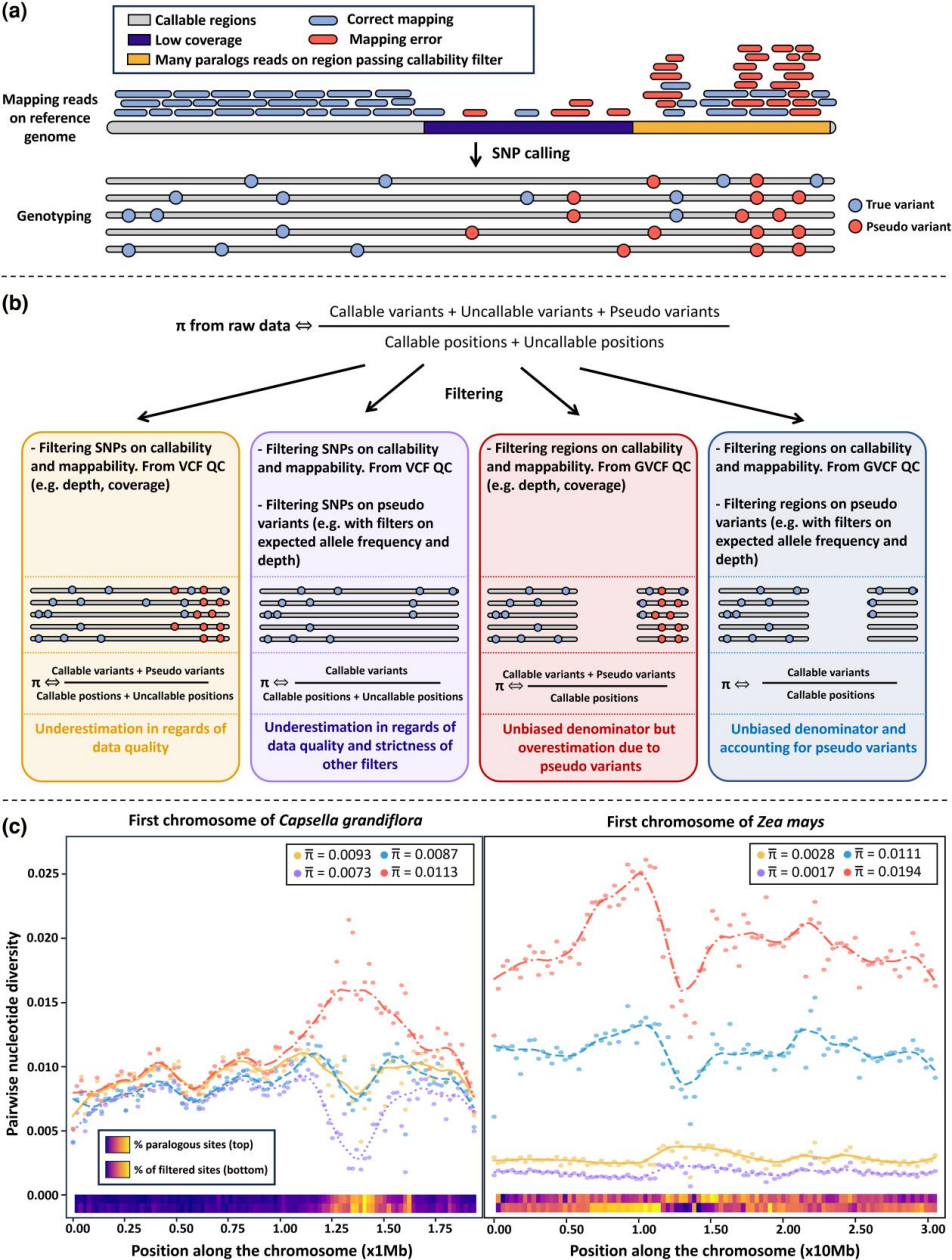
 Panel (a) shows the schematic consequences of mapping and genotyping; Panel (b) shows the theoretical filtering approaches; Panel (c) illustrates the consequences of filtering and scaling strategies on the estimation of π on the first chromosome of *Capsella grandiflora* and *Zea mays* (see Text S2 for method). The first filtering strategy (panel B in yellow) corresponds to a standard filtering of variant sites, based on coverage, depth, and GATK best practices (Van Der Auwera and O'Connor 2020, Text S2). This filtering only changes the numerator of the π formula, assuming all sites have been called. As a comparison, when the number of sites (denominator) is downscaled by removing uncalled regions, the corrected π increases, especially in regions with lower coverage where many uncalled sites are effectively removed from the denominator (panel c in red). However, genotype datasets contain artefactual variation (pseudo-variants) that bias further estimates of genetic diversity, especially for WGS data. The comparison between blue and red treatment highlights well this effect. For both species, average pi is more than doubled in red curve, in regions showing a high proportion of paralogs. With ngsparalogs, in *C. grandiflora* 22% variants were identified as paralogous sites and 32% in *Z. mays*. Finally, blue curve in Panel c shows that both rescaling the denominator by filtering non-callable regions and filtering pseudo-variant sites must be combined to achieve a most reliable estimate of π. Panel c also shows that the strength of the bias depends on the quality of the dataset such as genome coverage (93% for *C. grandiflora*, 78% for *Z. mays*) or sequencing depth (42 × for *C. grandiflora*, 7 × for *Z. mays), and its variation along chromosomes (*e.g. *stronger bias in centromeric regions)*. On *C. grandiflora,* final filtering (blue) is not so different than biased filtering (yellow) and does not affect average pi. In *Z. mays* however, because of lower depth and coverage mostly, average pi is 5 times larger (see Table S4 for other mean statistics).

**Fig. 3.**
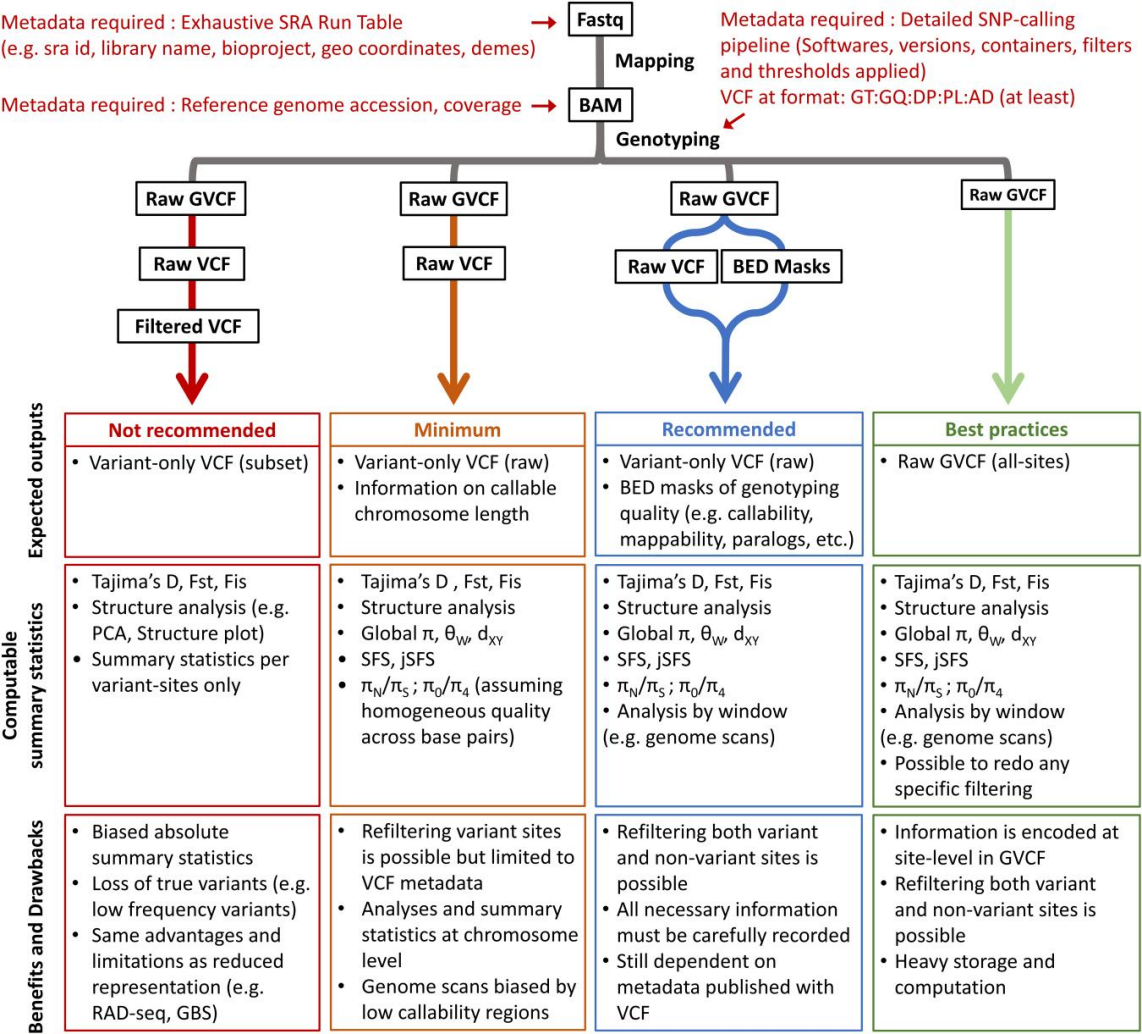
Graphical chart of the different strategies used to publish population genomics data. The recommended data workflow has the benefit of dealing with a variant-only raw VCF plus all necessary metadata in BED format, which makes it reusable with most software and easy to share. The best practice is to publish the raw all-sites GVCF for complete FAIRness and we advise it should become a gold standard to achieve. format in the coming years.

Variant filtering decisions motivated by quality control or computational convenience can strongly affect both genome-wide and local estimates of π and related statistics (see Box 1). Removing low-quality reads before mapping is a standard preliminary step, whereas filtering SNPs to mitigate mapping artifacts is less standardized across studies. This latter step plays an essential role in minimizing false positives: misalignment, copy number variants (CNVs), and paralogous regions absent from the reference genome all introduce an excess of heterozygosity and spurious intermediate-frequency alleles (Box 1, McKinney et al. 2017; Dallaire et al 2023; Jaegle et al. 2023). Given recent evidence for the abundance of CNVs in genomes of eukaryotic species, we suggest that filtering paralog-like errors should be a routine step in population genomics. A basic minimum approach would be to flag loci with excess heterozygosity, excess, and allelic imbalance coverage. In addition, several tools—e.g. paramask, ngsparalog, CHALLENGER, rCNV, Hdplot and mosdepth—have recently been developed specifically to identify and exclude problematic loci or uncallable regions (McKinney et al. 2017; Pedersen and Quinlan 2018; Dallaire et al. 2023; Karunarathne et al. 2023; Tjeng et al. 2025; Yilmaz et al. 2025). In any case, it is important to provide the information needed to allow other users to do their own filtering. Another filtering step influencing summary statistics estimation is post-variant filtering, such as Minor Allele Frequency (MAF) cutoffs or Linkage Disequilibrium (LD) pruning to eliminate non-independent SNPs. Although these filters are required for specific analysis, they introduce ascertainment bias for many other analyses and should not be used by default. Whatever the filtering strategy, it is crucial for comparability across studies to report at each filtering step the number of variants that pass filters, and to propagate the information on callable regions throughout the data workflow (Hemstrom et al. 2024).

## Some Guidelines and Best Practices

In this perspective, we have focused our recommendations on handling polymorphism data after genotyping, yet we are aware that such outputs can only be standardized if sequencing, alignment and genotyping methods yield comparable data. Unfortunately, the choice of both alignment and genotyping software are known to affect the resulting variant calls (Hatem et al. 2013; Vallebueno-Estrada and Swarts 2023). Additionally, variance in the magnitude of reference bias is also expected to make comparison of datasets more difficult (Brandt et al. 2015). These sources of bias will certainly limit the accuracy of comparisons between datasets from different studies, yet, in practice, we expect that these biases will often be small compared to real biological variation among species and populations. The current improvement in assembling standardized genomes (Larivière et al. 2024) and the development of pangenomic tools to account for structural diversity should help solve this issue, with the limitation that these methods are tested on model species (Secomandi et al. 2025). Resolving these issues, as well those discussed above, remains an open challenge that is beyond the scope of this paper. Here we suggest using strict FAIR principles for any genomic workflow, following our recommendations (Fig. 3, Box 1), to be able to reuse studies and reducing the amount of data regenerated and computing resources. Ensuring that population genetics data are *findable* and *accessible* is the first and most important step in enabling their reuse. We propose that population genetics summary statistics should be systematically and clearly presented in the main text or in Supplementary Tables, while more exhaustive datasets and associated metadata should be deposited in indexed, open-access repositories with a permanent DOI and in machine-readable open formats (such as csv or yaml). Song et al. 2023 recently evaluated the accessibility of WGS data in flowering plants; they show that among 294 studies, only 53 have a valid link to download a VCF and each has a specific filtering that makes them incomparable. To mitigate this issue, editors could systematically ask for basic summary statistics and metadata to be reported. As a starting point, we propose a standard workflow and a list of basic statistics that should be systematically reported in any population genomic studies (Fig. 3, Text S3).

Providing access to the processed data (i.e. VCF files), along with detailed metadata, is necessary for data reuse and reanalysis. A single validated protocol will probably never be met, because data processing strategies differ between research questions and the type of sequencing data (e.g. long-reads, short-reads, low coverage, RAD-seq). However, it is important to release data as open and untransformed as possible. Instead of the regular VCF file format (see its limitations in previous section), we recommend the genomic-aware gVCF specification (*GVCF—Genomic Variant Call Format* 2024), which fills almost all these limitations by allowing extra information for invariant sites. It has records for all sites called (GATK option “--output_mode EMIT_ALL_SITES”, FreeBayes option “--gvcf”), allowing to recover missing sites, and it must contain an accurate estimate of confidence in both the alternative and reference alleles at each site, even for reference homozygotes, in order to be kept all along the analysis workflow (e.g. for later joint genotyping of different datasets). In addition, its capacity to store metadata at the site level is easily extended with custom “INFO” and “FORMAT” fields (e.g. with bcftools and pysam, (Danecek et al. 2021; Pysam 2025)). However, gVCF files can be cumbersome to share and handle, a lighter alternative is to provide unfiltered VCF files with additional metadata (Beier et al. 2022), associated with reports of filtering strategies to be leveraged in new analyses (e.g. callability maps, paralog maps, SNP and/or individual black/white lists, see Table S3 for a more exhaustive list), as implemented in SNPArcher (Mirchandani et al. 2025). As discussed above, we encourage to add detection of paralogs as a routine step in the SNP-calling workflow (Box 1). All these steps can be done during SNP calling and are easy to store as CSV or BED files (see Fig. 3, recommended pipeline). Beyond considerations related to file format, software developers must ensure that they fully comply with these specifications in order to avoid any implicit assumptions about missing data and should explicitly enforce providing invariant and missing positions.

To conclude, recent initiatives like SORTEE guidelines in ecology and evolution (Pick et al. 2026), the GWAS catalogue (MacArthur et al. 2021; Hayhurst et al. 2022) or the “Comparative Population Genomics Data” (Mirchandani et al. 2024) provide promising examples of standardized frameworks for data and code quality control. As a possible starting point for population genomic data, we provide a tentative template for what self-assessment and editorial evaluation reports could look like (Texts S3 and S4). Overall, we advocate for the submission of summary statistics and metadata to become a routine for every new genomic dataset, similar to raw sequencing data deposition. It would alleviate the computational burden and carbon footprint of re-computing from raw sequencing, and hopefully lead to large-scale open databases, either based on summary statistics directly reported in the text or public VCF databases (Song et al. 2018; Cezard et al. 2022; Mirchandani et al. 2024), as it has been done with microsatellite data (Yashima et Innan 2017; Lawrence et al. 2019; Csilléry et al. 2025).

## Supplementary Material

evag103_Supplementary_Data

## Data Availability

All scripts and data necessary to reproduce this study are available at the InDoRES repository https://doi.org/10.48579/PRO/YWVYCY.
