# Supplementary material for: A Recipe for a Good π. How to Properly Estimate Population Genetics Summary Statistics and Why we Should Systematically Report Them
Source: Genome Biol Evol. 2026 Jun 5;18(6):evag103. doi: 10.1093/gbe/evag103 (PMC13236723; doi:10.1093/gbe/evag103)
Supplement: evag103_Supplementary_Data [file evag103_supplementary_data.zip › Supp_Table_3.docx]

| **Statistics** | **Generality** | **Input type** | **Possible errors and pitfalls** |
| --- | --- | --- | --- |
| **π** | Always | gVCF (or VCF + BED) | Wrong SNP calling (e.g. paralogs). Incorrect scaling (wrong denominator) |
| **θ_Watterson_** | Always | gVCF (or VCF + BED) | Wrong SNP calling (e.g. paralogs). Incorrect scaling (wrong denominator) |
| **F_IS_** | Always | VCF | Wrong SNP calling (e.g. paralogs). |
| **Tajima’s D** | Always | gVCF (or VCF + BED) | Wrong SNP calling (e.g. paralogs). |
| **π_N_/π_S_  or π_0_/π_4_** | If CDS regions | gVCF (or VCF + BED) + genome annotation | Wrong SNP calling (e.g. paralogs). Different choices for the number of S and NS "positions". Pi0/Pi4 is more straightforward. |
| **Pairwise F_ST_ matrix** | If two or more populations | VCF | Wrong SNP calling (e.g. paralogs). Incorrect scaling (wrong denominator) |
| **d_XY_** | If two or more populations | gVCF (or VCF + BED) | Wrong SNP calling (e.g. paralogs). |
| **SFS/jSFS** | Always | gVCF (or VCF + BED) | Wrong SNP calling (e.g. paralogs). Sensible to filters on allele frequencies |

| **Metadata** | **Output type** | **Utility** |
| --- | --- | --- |
| **Reference genome accession** | Fasta | Spatial reference for the position of sites along the genome. |
| **Raw genotypes** | gVCF or VCF (FORMAT=GT:AD:DP:GQ) | Allows reproducibility, comparability, different filtering and additional quality control. |
| **Number of bases called** | gVCF of BED | Allows to correct/rescale estimates per bp to properly account for missing sites. |
| **Repeat mask** | BED | Allows reproducibility and properly accounting for filtered sites. |
| **Mappability / Callability map** | BED | An alternative to the gVCF. Allows reproducibility and properly accounting for filtered sites. |
| **White/black list (e.g. paralogs)** | BED | Allows different filtering and assessing the effect of filters. |
| **Explicit sample size** | e.g. SRA Run Table | Can give a first idea of population structure if enough details (e.g. geographical coordinates). |

**Table 3: *(1)*** *Statistics of interest that should be included in the main text of any population genomic manuscript.* ***(2)*** *Metadata that should be linked to the paper in order for the data to be FAIR.*
