# Supplementary material for: A Recipe for a Good π. How to Properly Estimate Population Genetics Summary Statistics and Why we Should Systematically Report Them
Source: Genome Biol Evol. 2026 Jun 5;18(6):evag103. doi: 10.1093/gbe/evag103 (PMC13236723; doi:10.1093/gbe/evag103)
Supplement: evag103_Supplementary_Data [file evag103_supplementary_data.zip › Supp_text_1_2.docx]

Supplementary material

# Supplementary Text 1: Method for literature survey

## GBE articles

All studies published in GBE since 2009 (first year of publication) and indexed in Pubmed with the keywords “population genetics” or “population genomics” have been retrieved and analysed (n = 201 studies, Table S1). After a first filtering (keeping only empirical results and descriptions of newly produced dataset), we kept 113 studies for which we considered it was relevant to estimate standard population genetics summary statistics. For each paper, we looked if raw statistics are given in machine-readable format (i.e. not in a Figure), either in main text or in supplementary text or tables. We also noted for each paper if genomic metadata are linked to the paper. For the generation of Figure 1, we classified papers by data type under 4 categories: WGS, Sanger, RNAseq and Reduced representation which gather RADseq, GBS, SNP arrays and SNP chips. For panel A, we filtered out studies about Reduced representation as it is impossible to measure absolute statistics with this kind of data. For Fst statistics, we kept as relevant only papers with at least 2 populations. (See Table S1 for details).

## Maize datasets

### **Literature survey and choice of articles**

Using google scholar, we retrieved articles with the keywords (“Maize” OR “Zea”) AND (“Genetic diversity” OR “Domestication”). We then surveyed the articles and extracted those presenting population genetic analyses on wild and/or landrace and/or elite populations. We excluded articles focusing on experimental or restricted populations and those focusing only targeted sets of genes (ex: domestication genes, genes with specific functions). We ended up with 16 articles. We probably missed some studies but the aim was to illustrate the possible issues not to perform a comprehensive analysis.

### **Interpretation of outlier values (see Figure 2 and Table S2)**

Articles using GBS data

In Kumar et al. 2022, Dominguez et al. 2024 and Mariani-Zeffa et al. 2025 the values reported as π or nucleotide diversity range from 0.173 to 0.523, which are much too high for true π values but which are compatible with averaging over polymorphic SNPs only. In other publications, such statistics are reported as He (expected heterozygosity), which avoids confusion.

In Riveira-Rodriguez et al. 2023 the reported value is much too low: 2 × 10^−6^, roughly a factor 5000 compared to other studies on Teosinte. A possible explanation of this scaling issue is that the average nucleotide diversity computed on a filtered subset of SNPs (33,929) has been normalized by the full genome length. 33,929 SNPs on 3,604 individuals (as reported) gives a Waterson’s theta of 3587. Assuming a genome size of 218,2075,994 bp (reference genome), it gives a theta per site of θ = 1.64 × 10^−6^, which is compatible with the reported π value of 2 × 10^−6^.

Ma et al. 2025

The values of genetic diversity given in the article (π between 6.07 × 10^−4^ and 6.61 × 10^−4^) seems to be underestimated by a factor ten, compared to other studies. In the main text, it is reported that 124,726,264 SNPs on 100 individuals were used, giving a Watterson’s theta of 24,090,626. Assuming a genome size of 218,2075,994 bp (reference genome) with 98.68% coverage (as reported), it gives a theta per site of θ = 0.0098 for the whole data set. Given Fst values between 0.21 and 0.44 (as reported) with the approximate rule of thumb that θ_pop_ = θ_tot_ (1 - Fst), the θ_pop_ should range between 0.0055 and 0.0078, which would be compatible with Tajima’s π values between 6.07 × 10^−3^ and 6.61 × 10^−3^.

Comparison of Hufford et al. 2012 and Beissinger et al. 2016

These two studies are similar, comparing Teosinte and landraces, with similar sample scheme and sequencing approach, and partly performed by the same research groups. The reported values for genic regions are very similar for both teosinte and landraces as expected. However, for the total data set (more precisely intergenic for Beissinger et al. 2016 and total for Hufford et al. 2012, but genic regions only represent a few percent of the genome) there is a factor 1.4 for landraces and 1.95 for teosinte. Moreover, in Beissinger et al. 2016 π is higher in intergenic than in genic regions, as expected, whereas π is higher in genic regions, in Hufford et al. 2012, contrary to expectation. In the supplementary material of Hufford et al. 2012 it is indicated that they “observed higher coverage in more genic regions and lower coverage in regions rich in transposable elements”. It is thus likely that SNPs callability was lower in non-genic regions but that π was normalized by the total size of each region instead of size of the mappable and callable region.

# Supplementary Text 2: Material and Methods for the Box 1.

**Sampling**

We illustrated the biases on pi estimation on a Greek population of the annual, self-incompatible plant species *Capsella grandiflora* (Josephs et al. 2015, 78 samples from bioproject PRJNA275635) and on a population of *Zea mays subsp. parviglumis* (Valdivia et al. 2025 25 samples from bioproject PRJNA641489). (see detailed sampling in <https://doi.org/10.48579/PRO/YWVYCY>).

**Alignment and SNP Calling**

We started from raw fastq and used snpArcher (Mirchandani et al. 2024) to generate a raw VCF. We used this complete pipeline to map reads onto the reference genome of *Capsella rubella* (GCA_000375325.1) and *Zea mays* (GCF_902167145.1) with bwa (Li 2013) and to do the SNP calling with GATK with default parameters (following GATK best practices) (McKenna et al. 2010). In addition to the standard pipeline, we added a module to retrieve the information of the callability by site and by sample using mosdepth (Pedersen & Quinlan 2018). Each genotype above an absolute coverage threshold (here 10) and below a relative threshold (here 3 times sample-specific mean coverage) are kept in a BED file. We also added a module to detect paralogous SNPs, using ngsParalogs (Linderoth 2018) and default parameters. This tool leverages the allelic frequency and coverage of a SNP to calculate the likelihood of being artefactually polymorphic because of paralogy. This provided us with a black list of paralogous sites in a bed file format.

**Filtering**

To save storage and allow reproducibility, we always worked from the raw VCF on which we applied filtering with BED files. This allows the user to reproduce every filter we present but also try new ones. From the BED file giving callability and mappability by sites and by sample (i.e. for each genotype) we used a home-made script to set each genotype not in this file as missing in the VCF. From this new VCF, we measured pi by sites with VCFTools --sites-pi (Danececk et al. 2011). To illustrate the biases induced by not filtering for paralogs and second by not rescaling the effective callable chromosome length, we did four filters corresponding to the four combinations (see Figure 3, Box 1). Every filter has been made with BEDTools commands (Quinlan & Hall 2010) and consists in merging BED files together. To determine the threshold when a site should not be taken (i.e. how much missing genotype per site) we ran easySFS to make a projection of how many SNPs will be left if we remove a given number of genotypes and maximize the retained information. In *C. grandiflora*, we kept only sites that were called in at least 73 individuals out of 78 whereas in *Z. mays,* we kept sites called in at least 11 individuals out of 25.

**Estimation of statistics**

We used a homemade python script to measure π, Watterson’s θ and Tajima’s D by window based on the same correction used in pixy (Korunes & Samuk 2021). With the difference that we used a “variant-only” VCF plus BED files instead of an “all-sites” VCF as in pixy (see notebooks in published code for details). To measure π0/π4 ratio in *C. grandiflora*, we identified 0 and 4 degenerated sites with degenotate (Mirchandani et al. 2024) from the annotation of *C. rubella* (GCA_000375325.1). All statistics are available on the online repository (<https://doi.org/10.48579/PRO/YWVYCY>) as TSV tables or BED files.
