# Supplementary material for: A Recipe for a Good π. How to Properly Estimate Population Genetics Summary Statistics and Why we Should Systematically Report Them
Source: Genome Biol Evol. 2026 Jun 5;18(6):evag103. doi: 10.1093/gbe/evag103 (PMC13236723; doi:10.1093/gbe/evag103)
Supplement: evag103_Supplementary_Data [file evag103_supplementary_data.zip › Supp_text_3.docx]

# Supplementary Text 3

# Data & Code Editing Checklist: Genome Biology and Evolution

**Title:** Title of the study.

**Authors:** Authors names

**Date submitted:**

**Preprint :** <https://doi.org/xxxx/xxxxxx>

**Data & Code :** <https://doi.org/xxxx/xxxxxx>

**Editors:**

## **Detailed metadata are provided with the data**

- README (the content of directories and file is detailed)
- Sampling (e.g. IDs, species names, number, coordinates)
- Sequencing technology
- Reference genome accession (e.g. GCA, GCF accession)
- Raw sequencing data accessions (SRA/ENA accession)
- Number/type of markers
- Metadata is adequate (including README file)

## **Methods**

- Report software used to call variants
- Report software used to estimate summary statistics
- Software versions and options clearly presented in the manuscript/supplementary
- Code to reproduce filtering from raw data are provided

## **Summary statistics**

If relevant, summary statistics are estimated and presented in the main text or supplementary materials:

- π
- θ_W_
- Tajima’s D
- F_ST_
- D_XY_
- Uncertainty estimates are provided (e.g. s.e.m. or confidence intervals)
- Filtering process used to estimate statistics is reported
- Number of samples effectively used for population genetics analyses is easily findable
- Number of markers effectively used for population genetics analyses is easily findable
- Statistics presented in figures are provided somewhere else in machine-readable format (e.g. in CSV table)

## **Archived data is findable**

- Sequencing data accessible in a public database (e.g. NCBI SRA, ENA)
- (g)VCF and associated metadata have a DOI
- (g)VCF and associated metadata are cited in the manuscript
- (g)VCF and associated metadata have an appropriate license
- (g)VCF and associated metadata are open access and accessible

## **Archived data is interoperable**

- (g)VCF follows standard (g)VCF specifications
- (g)VCF is unfiltered
- (g)VCF has INFO/FORMAT fields (GT:AD:DP:GQ) to allow re-filtering
- Number of individuals and sites in (g)VCF correspond with what is reported in the manuscript
- BED files for filtering are provided
- Associated genomic maps and metadata (e.g. coverage, callability) are provided in standard open formats (e.g. BED, CSV, TSV)

## **Issues and suggestions :**
