# Supplementary material for: A Recipe for a Good π. How to Properly Estimate Population Genetics Summary Statistics and Why we Should Systematically Report Them
Source: Genome Biol Evol. 2026 Jun 5;18(6):evag103. doi: 10.1093/gbe/evag103 (PMC13236723; doi:10.1093/gbe/evag103)
Supplement: evag103_Supplementary_Data [file evag103_supplementary_data.zip › Supp_text_4.docx]

# Supplementary Text 4

# Self-assessment report

**Self-assessment questionnaire regarding FAIRisation of population genetic data, scripts, and code. To avoid frequent errors that hinder FAIRness of population genetics statistics :**

- Are the estimates, figures, and tables presented in the manuscript easily findable and accessible in a machine-readable format (e.g. a CSV table in supplementary, in opposition to Manhattan plots) ?
- Are population genomic data (raw reads and genotypes, e.g. VCF) and scripts to reproduce variant calling and downstream population genetics analyses provided in an open access repository or appropriate public database ?
- Can the population genomic data and scripts be accessed via the links provided in the submission form or directly within the manuscript?
- Are all relevant population genetics summary statistics (π, θ_W_, Tajima’s D, F_ST_, D_XY_) presented in the manuscript and/or supplementary materials ? Is the method to estimate summary statistics detailed and complete ?
- Are filtering strategies appropriate to summary statistics estimation ?
- Are data filtering and processing transparently described?
- Do population genomic data and scripts have an appropriate license allowing re-use (e.g., AGPL-v3, CC-BY)?
- Is the format of the data and scripts interoperable, i.e. they follow standard file formats and specifications, and provide metadata and software versions ? Are they annotated and linked to a README file comprehensible to a naive reader ?
- Do the estimates, figures, and tables generated from scripts and data in supplementary materials match all the estimates, figures, and tables presented in the manuscript?
